# Complete anterior segment reconstruction: Corneal transplantation and implantation of an iris prosthesis and IOL in a single surgery

**DOI:** 10.1177/1120672121991052

**Published:** 2021-01-28

**Authors:** Christian Mayer, Isabella Diana Baur, Julia Storr, Ramin Khoramnia

**Affiliations:** 1 Department of Ophthalmology, University of Heidelberg, Heidelberg, Germany; 2 Ophthalmology Clinic and Polyclinic, Technical University of Munich, Munich, Germany

**Keywords:** Trauma, corneal transplantation, cornea/external disease, IOLs, lens/cataract, secondary IOL implantation, penetrating keratoplasty

## Abstract

**Purpose:**

Patients who have suffered an ocular trauma may present with varying degrees of injury to the anterior segment. In this retrospective interventional case series, we report the outcome of seven patients who underwent complete anterior segment reconstruction in a single surgery.

**Methods:**

All patients with posttraumatic corneal decompensation or scar, aphakia, and iris defect underwent human donor corneal graft transplantation and implantation of an intraocular lens combined with a flexible silicone iris prosthesis. Postoperative examinations included assessment of best corrected distance visual acuity, objective refraction, and intraocular pressure. Sensitivity to glare and subjective discontent with the eye’s appearance was rated on a scale from 1 to 10, with 1 standing for low and 10 for high severity.

**Results:**

Mean best corrected distance visual acuity (BCDVA) was 1.51 ± 0.26 logMAR preoperatively and 1.29 ± 0.36 logMAR postoperatively. Mean IOP was 15.71 ± 8.94 mmHg pre-surgery and 13.57 ± 6.52 mmHg post-surgery. The mean sensitivity to glare was reduced from 7.17 ± 2.91 to 3.80 ± 3.43 and subjective cosmetic disfigurement was reduced from 5.33 ± 3.35 to 1.80 ± 1.60.

**Conclusions:**

A single surgery technique for entire anterior segment reconstruction in trauma patients can effectively reduce glare and patient discontent with the eye’s appearance.

## Introduction

Traumatized eyes often show multiple structural damages. In many cases, almost the complete anterior segment of the eye is affected, including cornea, iris, and lens. Most of the injuries result from foreign material that hits the eye from anterior, with only the eyelid protecting the globe. Due to the direction of the impact, anatomical structures are involved consecutively in the following order: first the cornea, secondly the iris, lastly the lens. In even more severe cases, also the posterior segment of the eye is affected.^1^ It is sometimes challenging to reconstruct these structures in one single surgery.

However, nowadays, there are implants which allow surgeons to reconstruct anterior ocular structures: human donor corneal grafts or corneal prostheses^2^ can substitute the cornea, the human lens can be replaced with an intraocular lens, and the human iris can be substituted with an iris prosthesis.^3^ We present our results in trauma cases in which there is a complete reconstruction of the anterior segment – with corneal transplantation, implantation of an iris prosthesis, and implantation of an IOL – all performed in one single surgery.

## Material and methods

Inclusion criteria were (1) the need for a corneal transplant, IOL, and iris reconstruction, (2) visual acuity of at least light perception, and (3) stable posterior segment with an attached retina. Eight patients were included in this retrospective, interventional case series. From eight cases, outcomes can be reported for seven patients. One patient was excluded from the investigation after failure to attend eye-examinations. Patient consent to publish this case series including images was obtained.

Written informed consent was obtained, and the patient was especially informed about the long surgery time and the risk for intra- and postoperative complications (e.g. retinal detachment, IOP rise, and corneal graft failure). The study was approved by the local Ethics Committee and made under the tenets of the Declaration of Helsinki. The corneal donations were obtained from the responsible eye bank, no tissues were procured from vulnerable populations, for example prisoners.

### Material

**Figure 1.**
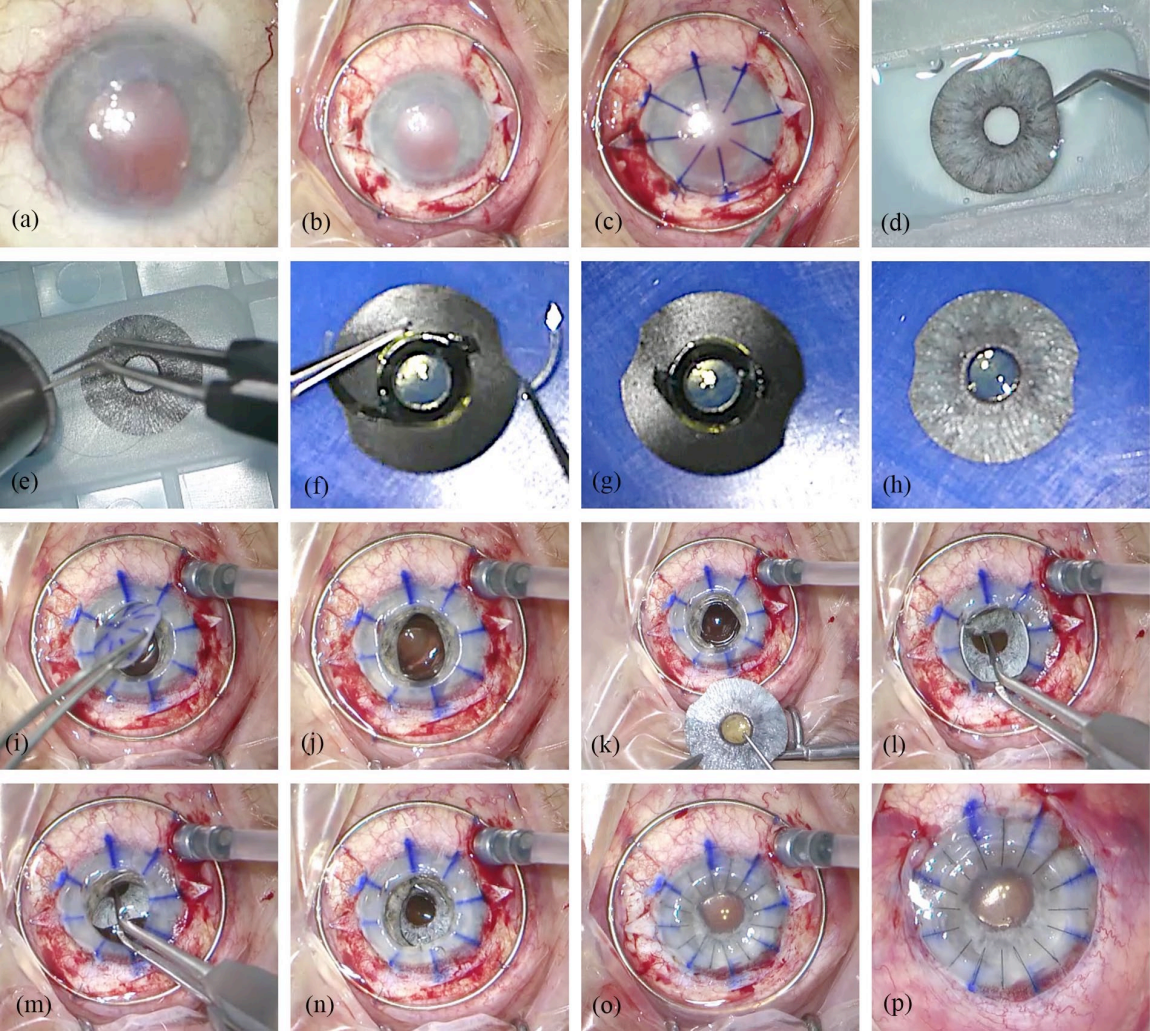
Surgical steps: (a) anterior segment at the beginning of surgery shows corneal scarring, traumatic iris defect mydriasis, and aphakia, (b) suturing of the Flieringa ring onto the sclera and preparation of the scleral flaps at the 3 and 9 o’clock position, (c) corneal marking, (d) ArtificialIris with individually colored anterior surface, (e) trephination of the iris prosthesis, (f) suturing of the IOL on the backside of the iris prosthesis, (g) cutting of the IOL haptics, (h) photo of the combined ArtificialIris-IOL-“sandwich,” (i) trephination and removal of the altered cornea, (j) open sky, (k) placement of the sutures for scleral fixation through the central opening beneath the scleral flaps, (l) and (m) implantation of the ArtificialIris-IOL-“sandwich” through the large central opening, (n) placement and fixation of the combined implant in the ciliary sulcus by tightening of the sutures, (o) insertion and suturing of the corneal graft, and (p) anterior segment at the end of surgery.

1. The corneal grafts were standard not HLA-matched human grafts. Therefore, we depended on the availability of human donor donations. Single continuous sutures, double-running cross-stich sutures (according to Hoffmann) or single interrupted sutures were used for fixation of the graft.
2. For treatment of aphakia or traumatic cataract, we used the standard IOL used at our center for routine patients with aphakia. In patients who were already pseudophakic, the IOL was explanted and replaced. We chose a flexible acrylic IOL, the Aspira MC6125AS-Y (HumanOptics, Erlangen, Germany), which because of the soft material is amenable for suturing and its haptics can be readily severed.
3. We used the flexible, ArtificialIris (AI) implant (HumanOptics, Erlangen, Germany, Figure 1(d)), which features an overall diameter of 12.8 mm and a fixed pupil aperture of 3.35 mm. The thickness decreases from the pupillary margin (0.4 mm) to the peripheral edge (0.25 mm). The surface of the prosthesis is individually handcrafted with colored silicone, based on a photographic documentation of the patient’s iris in the healthy fellow eye. The posterior consists of a smooth, opaque, and black silicone layer. This device received European Conformity (CE) marking for use within the European Economic Area in 2011, and Food and Drug Administration (FDA) approval in the United States in 2018. One advantage of this silicone-iris device is that most commercially available IOLs can be sutured to its posterior side (Figure 1(f)—(h)).

Description of surgical procedure (Figure 1, Supplemental Video):

All patients were aphakic, pseudophakic or had traumatic cataract as well as a traumatic iris defect and corneal decompensation or corneal scar after ocular trauma.

All seven reconstructive procedures were performed with general anesthesia and by the same surgeon (CM). All procedures occurred a sufficiently long time after the initial trauma in an interval in which the eye was free of inflammation and in a stable condition. The time between the trauma and the reconstructive surgery varied between 9 months and 37 years. None of the surgeries were performed as primary repair of the trauma.

After opening of the conjunctiva, a Flieringa ring was sutured onto the sclera for intra-operative stabilization of the globe. Scleral flaps were prepared in the 3 and 9 o’clock position. After trephination of the cornea with handheld trephines, the tissue bridges were dissected with Troutman corneal scissors. In pseudophakic patients, the IOL was removed through the opening of the trephined cornea. In patients with traumatic cataract, we proceeded with capsulorhexis and phacoemulsification. The AI was trephined to the required diameter and two prophylactic iridectomies were made on the prosthesis. We used 9.0 or 10.0 polypropylene sutures to attach the AI to the IOL. These sutures penetrated the IOL haptic at a point close to the optic-haptic junction from the posterior direction; then the IOL and AI together were turned around on the front side to go back through the iris. A knot was placed on the posterior side. To reduce the size of the AI and IOL combination and to keep the incision size as small as possible, the distal part of the IOL haptics were severed with surgical scissors. The sutures fixing the combined implants to the sclera were then attached at the 3 and 9 o’clock positions, making sure to set the attachment points in opposing directions and thus achieve a well centered pupil. The combined AI + IOL was then inserted as a folded “sandwich” into the eye through the large opening in the trephined cornea (open-sky-procedure). The combined implants were positioned in the ciliary sulcus and attached with 9.0 or 10.0 polypropylene sutures onto the sclera. In the final step, the corneal graft was firmly sutured with 10.0 nylon sutures.

## Follow-up and ocular examination

Main outcome measures were subjective impairment from glare, subjective cosmetic disfigurement, patient satisfaction, and intraocular pressure (IOP). Best-corrected distance visual acuity (BCDVA) was only a secondary outcome measurement because of the recognition that long-term rehabilitation is required.

We measured BCDVA at every examination visit with a Snellen-chart at 6 m distance. Postoperative refraction was determined using objective refractometry.

For the subjective evaluation of the patients’ complaints and the evaluation of the postoperative result, we used a questionnaire in German language, that we designed specifically for use in patients who underwent AI implantation.

The first part of the questionnaire consists of two numerical rating scales from 1 to 10, with 1 standing for low and 10 for high severity, that allows the patient to rate their sensitivity to glare and their discontent with their eye’s appearance.

The second part of the questionnaire is composed of a numerical rating scale from 1 to 10, where 1 represents minimum satisfaction and 10 is maximum satisfaction, so that the patients can indicate their overall satisfaction.

We performed the first part of the assessment preoperatively and postoperatively and the second part of the questionnaire was used at the postoperative follow-up visits.

IOP was measured at every examination using Goldmann applanation tonometry.

## Results

Table 1 summarizes the patient characteristics and outcomes of the seven patients included in this study.

**Table 1.** Patient characteristics and outcomes.

Case		1	2	3	4	5	6	7
Corresp. Figures		2(a) to (d)	3(a) to (d)	3(e) to (h)	3(i) to (l)	3(m) to (p)	3(q) to (t)	3(u) to (x)
Sex		M	F	F	M	M	F	M
Age		66	56	34	31	59	41	51
Eye		OD	OS	OD	OD	OD	OD	OD
Additional pathologies		Glaucoma, zonulolysis, exotropia	Exotropia	Glaucoma, synechia, status post keratoplasty	Glaucoma, exotropia	Synechia, esotropia	Status post keratoplasty	Status post keratoplasty
Lens status pre surgery		Pseudophakic	Pseudophakic	Pseudophakic	Pseudophakic	Traumatic cataract	Pseudophakic	Aphakic
Reason for keratoplasty		Corneal decompensation	Corneal scarring	Corneal decompensation	Corneal scarring	Corneal scarring	Corneal decompensation	Corneal decompensation
Follow-up (months)		8	14	4	4	2	6	1
BCDVA	Pre-surgery	1.98	1.7	1.3	1.3	1.2	1.7	1.4
	Post-surgery	1.7	1.5	0.7	1.4	1.0	1.7	1.0
Postoperative refraction		+8.25/−4.0/65°	+2.5/−2.75/165°	−1.0/−3.0/120°	**–**	+3.75/−7.0/123°	+1.75/−7.0/155°	−5.25/−5.5/120°
IOP	Pre-surgery	11	21	16	35	9	10	8
	Post-surgery	18	20	24	11	6	7	9
Subjective impairment from glare	Pre-surgery	2	10	5	9	–	10	7
	Post-surgery	1	5	2	1	2	7	10
Subjective cosmetic disfigurement	Pre-surgery	1	6	10	6	–	1	8
	Post-surgery	1	1	5	1	2	4	1
Overall satisfaction		8	5	10	9	4	6	6
Postoperative complications		–	Repeated Ozurdex Implantation for postoperative macular edema	–	Ahmed Valve implantation	-	Retinal detachment requiring repeated surgery and silicone oil filling	–

**Figure 2.**
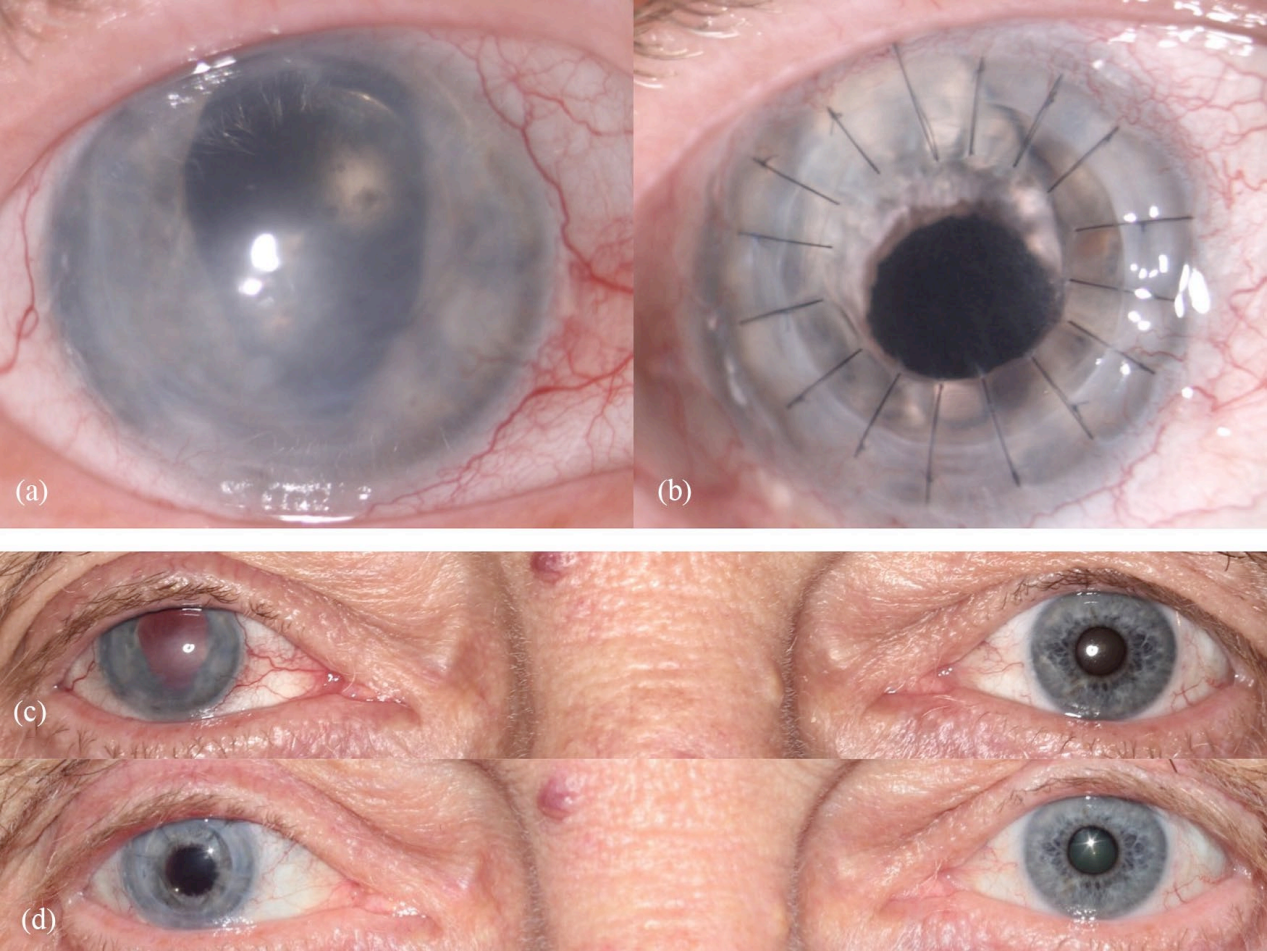
Findings in patient 1: (a) preoperative close-up photograph of the traumatized right eye, (b) postoperative close-up photo after combined surgery (perforating keratoplasty and implantation of an artificial iris and IOL), (c) preoperative binocular photograph, and (d) postoperative binocular photograph.

**Figure 3.**
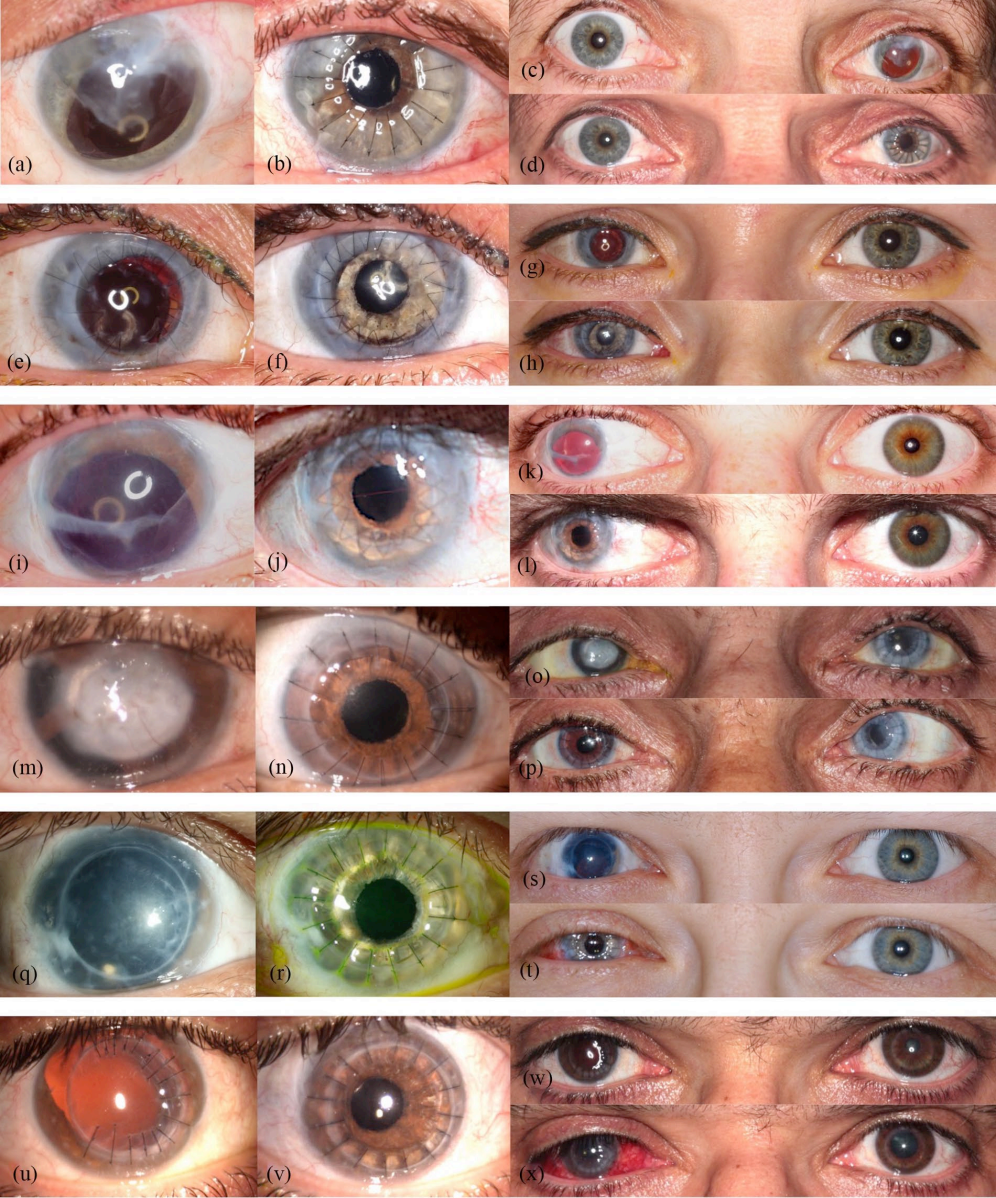
Preoperative close-up photographs of the traumatized eyes (left column) and respective postoperative close-up photographs of the same eyes (middle column) in the remaining six patients. Preoperative binocular photographs (upper sections of the right column) and postoperative binocular photographs (lower sections of the right column) ((a)–(x)).

Figure 2(a) and (c) and Figure 3 (left column) show the preoperative findings of all patients.

The mean follow-up time was 5.43 ± 4.24 months, ranging from 1 to 14 months. Mean BCDVA was 1.51 ± 0.26 logMAR preoperatively and 1.29 ± 0.36 logMAR postoperatively. BCDVA did not decrease in any patient. All patients had a considerable postoperative astigmatism. The median value for diopter cylinder was −4.75 D with a range from −2.75 to −7.0 D. Mean IOP was 15.71 ± 8.94 mmHg pre-surgery and 13.57 ± 6.52 mmHg post-surgery. Patients rated subjective visual impairment from glare 7.17 ± 2.91 on average preoperatively and 4.00 ± 3.21 on average postoperatively. Mean subjective cosmetic disfigurement was 5.33 ± 3.35 pre-surgery and 2.14 ± 1.55 post-surgery. The cosmetic result for each patient is shown in Figure 2(b) and (d) and Figure 3. Patients were very satisfied with the cosmetic result of surgery (Figure 2(d)). Mean patient satisfaction on the numerical scale was 6.86 ± 2.03.

There were no significant intraoperative complications. The postoperative complications observed are also listed in Table 1. One patient (Case 2) developed postoperative macular edema and was treated with four consecutive Ozurdex implantations. Another patient (Case 4) with pre-existing glaucoma needed glaucoma surgery for IOP control and was treated with an Ahmed valve implantation. In Case 6, a retinal detachment occurred 2 months postoperatively. The patient was treated with vitrectomy and C2F6 endotamponade but had a recurrent retinal detachment which was treated with silicone oil endotamponade. We observed that the new, iris-lens-diaphragm effectively prevented the migration of silicone oil into the anterior chamber. Dislocation of lens and/or artificial iris was not observed.

## Discussion

We report on the outcomes of seven cases of anterior segment reconstruction following traumatic injury. So far, only a few cases of combined keratoplasty, IOL and AI implantation have been published. Forlini et al.^4^ reported four cases of surgical correction of posttraumatic aphakia and aniridia out of which only two patients underwent keratoplasty in the same surgery session using a technique similar to the one described in this study. The authors used the same ArtificialIris that we used but different models of IOL, and they found a good visual rehabilitation as well as a satisfying cosmetic result. Yoeruek et al.^5^ treated five patients with combined AI + IOL implantation and keratoplasty. They observed an increase in BCDVA, reduction of glare sensitivity and a stable position of the implants in all cases. The postoperative complications included graft failure and IOP elevation in patients with pre-existing glaucoma.

Another approach to treat patients with corneal decompensation, aphakia, and iris defects would be the implantation of a standard iris reconstruction lens in combination with a corneal graft transplantation. An advantage of this approach would be the fact that the iris prosthesis does not have to be sutured to the IOL before the implantation and there is no need to adapt it in size. This method provides good functional results.^6–8^ However, these lenses are only available in black, for example the Morcher iris reconstruction lens (Morcher GmbH, Stuttgart, Germany), or in a limited number of colors, as for the Ophtec iris reconstruction lens (Ophtec B.V., Groningen, The Netherlands). Therefore, the result cannot be as esthetically pleasing as with the AI that we used, which is individually hand painted using a photograph to match the remaining iris tissue and/or the iris of the fellow eye. Furthermore, the Morcher aniridia implants are made of rigid poly ethyl methacrylate (PEMA) which would not be an ideal material for our technique. However, suturing of a PEMA IOL onto the sclera can also be done.

Penetrating keratoplasty in combination with cataract removal and IOL implantation is known as “triple procedure.” A problem in triple procedures is the refractive outcome. This is mostly due to unpredictable postoperative keratometry.^9,10^ It is postulated that a delayed sequential approach may be beneficial in terms of achieving the desired refractive outcome. However, contrary to what was expected, the refractive outcome after delayed sequential surgeries is not superior to the outcome after triple procedure and the triple procedure offers a faster visual rehabilitation.^11,12^ Therefore, the patient can benefit from a single procedure, because this minimizes the cumulative risk of several surgeries and the additional trauma to the corneal transplant.

The technique we used entails several challenges: Depending on the preexisting conditions, the occurrence of certain postoperative complications is more likely in patients with traumatized eyes. Traumas affecting the retina can lead to an increased permeability of the blood-retinal barrier, leading to a higher risk of macular edema.^13,14^ The risk of postoperative IOP elevation following other surgical procedures such as cataract surgery or vitrectomy is higher in glaucoma patients.^15–17^ The same applies to anterior segment reconstruction surgery. Furthermore, the varying degree of traumatized tissue makes it necessary to adapt the surgical procedure individually.

Another difficulty in combined AI and IOL implantation and keratoplasty, is the need to minimize the interaction of the AI + IOL combination with the donor graft. The combination needs to be attached in a stable position to prevent contact between it and the corneal graft, to avoid endothelial cell loss.

We used 9.0 or 10.0 polypropylene sutures to attach the AI to the IOL and to attach the implants onto the sclera. Gore-tex sutures proved to be a safe option for scleral fixation of IOLs^18^ and can be used alternatively for both purposes.

A number of factors can potentially hamper the achievement of the desired target refraction. Firstly, the effective lens position is difficult to predict. Also, biometry and thus IOL power calculation can be difficult in patients with corneal opacity. Optical biometry is superior to applanation ultrasound measurement,^19^ but it requires clear media and the patient being able to fixate: requirements which are often not met in patients with corneal decompensation. Then IOL power calculation has to be based on ultrasound measurement or, in severe cases, even by relying on the biometry of the fellow eye. After corneal transplantation, patients often have a considerable astigmatism.^20^ These factors contribute to a possible deviation from the target refraction. If there is a high astigmatism or total refractive error postoperatively, a contact lens can be fitted.^21^

AI implantation has proven to be an effective therapeutic option in patients with high glare sensitivity due to traumatic or atraumatic iris defects. An AI implantation significantly reduces the pupillary aperture and can therefore reduce the sensitivity to glare and improve contrast sensitivity.^22^ It also provides excellent aesthetic results.^23^ Therefore, the patient satisfaction with the functional and cosmetic result is very high,^22,24^ although in some cases a preoperative strabismus persisted postoperatively. While it is not a primary goal of AI implantation, BCDVA is reported to increase after the implantation.^3,25–28^ This is most likely due to simultaneous cataract surgery or secondary IOL implantation in patients with aphakia^29^ as well as due to the reduction of glare.

A number of reports of complications following AI implantation including decreased BCDVA, IOP elevation, AI dislocation or decentration, corneal decompensation, retinal detachment, and macular edema have been published.^25,26,30^ The postoperative complications that we observed in our patients included IOP elevation, macular edema, and retinal detachment. We saw a considerable reduction in visual impairment from glare and in subjective cosmetic disfigurement. BCDVA was stable or increased in all patients. We recorded a very high patient satisfaction with the procedure.

When there is residual natural iris tissue, hemorrhage of the remnant iris or darkening of the natural iris tissue can be observed.^25,26^ Another complication related to remaining natural iris tissue is the residual iris retraction syndrome (RITS), where in some patients with incomplete aniridia, the original pupillary aperture gradually enlarges over time after the AI implantation. This phenomenon is associated with complications like closed angle glaucoma and chronic inflammation. The cause of RITS is unknown.^31^

Similar considerations have to be taken into account for our procedure, penetrating keratoplasty combined with implantation of an AI/IOL complex. As previously mentioned, it is known from triple procedure that the refractive outcome is not strongly influenced by choice of a combined surgery versus sequential approach. The advantage of performing all in one single session is to minimize trauma to the corneal transplant. But another advantage is that the AI in combination with the IOL can be smoothly implanted through the large aperture in the cornea (open sky surgery). Thus a sclero-corneal tunnel incision is avoided and the patient need not return for a second surgery session.

One limitation of this study is the range in follow-up time. For one patient results can only be reported 1 month postoperatively. Longer Follow-up is required to evaluate whether the nature of post-operative complications will change over time.

Another limitation is the lack of a standardized questionnaire to evaluate the subjective impairment from glare and subjective cosmetic disfigurement. We used a questionnaire developed specifically for patients who underwent AI implantation in German language to offer our patients a questionnaire that is easy to understand and tailored to the key issues according to our clinical experience.

Our procedure allows achievement of a functional and aesthetically appealing good result for the patient. The use of an artificial iris, specifically designed for the patient, together with soft IOLs allow an individualized type of anterior segment reconstruction. We therefore recommend this single surgery technique as most feasible in the reconstruction of traumatized eyes with corneal scaring, large iris defects, and aphakia.

## References

[1] Eagling EM. Perforating injuries of the eye. Br J Ophthalmol 1976; 60: 732. 1009048 10.1136/bjo.60.11.732 PMC1042827

[2] Shalaby Bardan A Al Raqqad N Zarei-Ghanavati M , et al. The role of keratoprostheses. Eye (Lond) 2018; 32: 7–8. 29192685 10.1038/eye.2017.287 PMC5770726

[3] Spitzer MS Yoeruek E Leitritz MA , et al. A new technique for treating posttraumatic aniridia with aphakia: first results of haptic fixation of a foldable intraocular lens on a foldable and custom-tailored iris prosthesis. Arch Ophthal 2012; 130: 771–775. 22801839 10.1001/archophthalmol.2011.1778

[4] Forlini C Forlini M Rejdak R , et al. Simultaneous correction of post-traumatic aphakia and aniridia with the use of artificial iris and IOL implantation. Graefes Arch Clin Exp Ophthalmol 2013; 251(3): 667–675. 23324893 10.1007/s00417-012-2254-7

[5] Yoeruek E Bartz-Schmidt KU. A new knotless technique for combined transscleral fixation of artificial iris, posterior chamber intraocular lens, and penetrating keratoplasty. Eye 2019; 33: 358–362. 30209266 10.1038/s41433-018-0202-4 PMC6460692

[6] Mashor RS Bahar I Kaiserman I , et al. Combined penetrating keratoplasty and implantation of iris prosthesis intraocular lenses after ocular trauma. J Cataract Refract Surg 2011; 37(3): 582–587. 21333880 10.1016/j.jcrs.2010.10.038

[7] Phillips PM Shamie N Chen ES , et al. Transscleral sulcus fixation of a small-diameter iris-diaphragm intraocular lens in combined penetrating keratoplasty and cataract extraction for correction of traumatic cataract, aniridia, and corneal scarring. J Cataract Refract Surg 2008; 34: 2170–2173. 19027578 10.1016/j.jcrs.2008.06.048

[8] Miller AR Olson MD Miller KM. Functional and cosmetic outcomes of combined penetrating keratoplasty and iris reconstruction lens implantation in eyes with a history of trauma. J Cataract Refract Surg 2007; 33: 808–814. 17466853 10.1016/j.jcrs.2007.01.018

[9] Javadi M-A Feizi S Moein H-R. Simultaneous penetrating keratoplasty and cataract surgery. J Ophthalmic Vis Res 2013; 8(1): 39–46. 23825711 PMC3691977

[10] Inoue Y. Corneal triple procedure. Semin Ophthalmol 2001; 16(3): 113–118. 15513428 10.1076/soph.16.3.113.4202

[11] Cazabon S Quah SA Jones MN , et al. Sequential versus combined penetrating keratoplasty and cataract surgery. Optom Vis Sci 2010; 87: 482–486. 20473235 10.1097/OPX.0b013e3181e1728e

[12] Seitz B Langenbucher A Viestenz A , et al. Cataract and keratoplasty—simultaneous or sequential surgery? Klin Monbl Augenheilkd 2003; 220: 326–329. 12766821 10.1055/s-2003-39429

[13] Cunha-Vaz J . Mechanisms of retinal fluid accumulation and blood-retinal barrier breakdown. In: Coscas G Loewenstein A Cunha-Vaz J , et al. (eds) Macular edema. Basel, Switzerland: Karger Publishers, 2017, pp.11–20. 10.1159/000455265 28351041

[14] Cunha-Vaz J . Studies on the permeability of the blood-retinal barrier. II. Breakdown of the blood-retinal barrier by injury. Br J Ophthalmol 1966; 50: 454. 5915320 10.1136/bjo.50.8.454 PMC506253

[15] Annam K Chen AJ Lee IM , et al. Risk factors for early intraocular pressure elevation after cataract surgery in a cohort of United States veterans. Mil Med 2018; 183: e427–e433. 10.1093/milmed/usx113 29425312

[16] Grzybowski A Kanclerz P. Early postoperative intraocular pressure elevation following cataract surgery. Curr Opin Ophthalmol 2019; 30: 56–62. 30489361 10.1097/ICU.0000000000000545

[17] Jabbour E Azar G Antoun J , et al. Incidence and risk factors of ocular hypertension following pars plana vitrectomy and silicone oil injection. Ophthalmologica 2018; 240: 129–134. 30036875 10.1159/000489792

[18] Khan MA Gupta OP Smith RG , et al. Scleral fixation of intraocular lenses using Gore-Tex suture: clinical outcomes and safety profile. Br J Ophthalmol 2016; 100: 638–643. 26319945 10.1136/bjophthalmol-2015-306839

[19] Rose LT Moshegov CN. Comparison of the Zeiss IOLMaster and applanation A-scan ultrasound: biometry for intraocular lens calculation. Clin Exp Ophthalmol 2003; 31: 121–124. 12648044 10.1046/j.1442-9071.2003.00617.x

[20] Jensen AD Maumenee A. Refractive errors following keratoplasty. Trans Am Ophthalmol Soc 1974; 72: 123. 4618393 PMC1311391

[21] Szczotka LB Lindsay RG. Contact lens fitting following corneal graft surgery. Clin Exp Optom 2003; 86: 244–249. 12859244 10.1111/j.1444-0938.2003.tb03113.x

[22] Mayer CS Reznicek L Hoffmann AE. Pupillary reconstruction and outcome after artificial iris implantation. Ophthalmology 2016; 123: 1011–1018. 26935356 10.1016/j.ophtha.2016.01.026

[23] Yildirim TM Khoramnia R Masyk M , et al. Aesthetics of iris reconstruction with a custom-made artificial iris prosthesis. PloS One 2020; 15: e0237616. 32790803 10.1371/journal.pone.0237616 PMC7425955

[24] Mayer C Hoffmann A Prahs P , et al. Functional outcomes after combined iris and intraocular lens implantation in various iris and lens defects. BMC Ophthalmol 2020; 20(1): 370. 32933506 10.1186/s12886-020-01621-8 PMC7493881

[25] Rickmann A Szurman P Januschowski K , et al. Long-term results after artificial iris implantation in patients with aniridia. Graefes Arch Clin Exp Ophthalmol 2016; 254: 1419–1424. 26892143 10.1007/s00417-016-3292-3

[26] Mayer CS Laubichler AE Khoramnia R , et al. Challenges and complication management in novel artificial iris implantation. J Ophthalmol 2018; 2018: 3262068. 30345111 10.1155/2018/3262068 PMC6174745

[27] Mayer C Tandogan T Hoffmann AE , et al. Artificial iris implantation in various iris defects and lens conditions. J Cataract Refract Surg, 2017; 43(6): 724–731. 28732604 10.1016/j.jcrs.2017.06.003

[28] Mayer CS Baur I Storr J , et al. Surgical Management for Silicone Oil Barrier of Traumatic Aniridia with Aphakia: Suturing of Temporary Iris-Diaphragm Prior to Final Iris-Lens-Diaphragm Implantation. Clin Ophthal, 2020; 14: 4439–4450. 10.2147/OPTH.S284159 PMC7762446 33376298

[29] Mayer C Son H-S Labuz G , et al. In vitro optical quality assessment of a monofocal IOL sutured to an artificial iris. J Cataract Refract Surg 2020; 46: 1184–1188. 32818330 10.1097/j.jcrs.0000000000000287

[30] Spitzer MS Nessmann A Wagner J , et al. Customized humanoptics silicone iris prosthesis in eyes with posttraumatic iris loss: outcomes and complications. Acta Ophthalmol 2016; 94: 301–306. 26805757 10.1111/aos.12946

[31] Mayer CS Laubichler AE Masyk M , et al. Residual iris retraction syndrome after artificial iris implantation. Am J Ophthalmol 2019; 199: 159–166. 30236771 10.1016/j.ajo.2018.09.001

